# EEG Authentication System Based on One- and Multi-Class Machine Learning Classifiers

**DOI:** 10.3390/s23010186

**Published:** 2022-12-24

**Authors:** Luis Hernández-Álvarez, Elena Barbierato, Stefano Caputo, Lorenzo Mucchi, Luis Hernández Encinas

**Affiliations:** 1 Computer Security Lab, Universidad Carlos III de Madrid, 28911 Leganés, Spain; 2 Institute of Physical and Information Technologies, Spanish National Research Council, 28006 Madrid, Spain; 3 Department of Agriculture, Food, Environment and Forestry, University of Florence, 50144 Firenze, Italy; 4 Department of Information Engineering, University of Florence, 50139 Firenze, Italy

**Keywords:** electroencephalogram, machine learning, multi-class classifier, one-class classifiers, user authentication

## Abstract

In the current Information Age, it is usual to access our personal and professional information, such as bank account data or private documents, in a telematic manner. To ensure the privacy of this information, user authentication systems should be accurately developed. In this work, we focus on biometric authentication, as it depends on the user’s inherent characteristics and, therefore, offers personalized authentication systems. Specifically, we propose an electrocardiogram (EEG)-based user authentication system by employing One-Class and Multi-Class Machine Learning classifiers. In this sense, the main novelty of this article is the introduction of Isolation Forest and Local Outlier Factor classifiers as new tools for user authentication and the investigation of their suitability with EEG data. Additionally, we identify the EEG channels and brainwaves with greater contribution to the authentication and compare them with the traditional dimensionality reduction techniques, Principal Component Analysis, and $\chi^{2}$ statistical test. In our final proposal, we elaborate on a hybrid system resistant to random forgery attacks using an Isolation Forest and a Random Forest classifiers, obtaining a final accuracy of 82.3%, a precision of 91.1% and a recall of 75.3%.

## 1. Introduction

The recent development of technology has made it possible to implement sophisticated software even on small hardware. As a result, common devices such as smartphones, tablets, and smartwatches are typically equipped with many sensors and complex software. These portable devices now contain much of our digital lives, allowing us to access essential services on the go, such as private documents, e-payment, online shopping, and e-banking. In this context, user-friendly authentication systems that can be used frequently are essential for privacy and data protection [1]. These systems are often based on something the user knows (e.g., passwords or pins), owns (e.g., identification cards), or is (e.g., biometric data such as fingerprints) [2]. However, the first two approaches have disadvantages: complex passwords are difficult to remember, simple codes can be easily guessed by attackers, and identification objects can be physically stolen and grant permanent access after authentication [2]. The use of biometric data is preferable as it provides representative and unique information specific to each user’s body.

Biometric authentication systems often rely on artificial intelligence (AI) algorithms to differentiate specific users and prevent identity fraud [3]. AI algorithms are powerful tools that learn how to classify data based on their structure. To do this, they are trained and tested on data samples defined by biometric *features*. These features can be defined manually (using Machine Learning or ML) or extracted automatically (using Deep Learning or DL). Additionally, an AI model can be defined as multiclass (MC) if it learns from data with different labels (i.e., a classification problem) or as one-class (OC) if it learns from data from only one group (i.e., outlier detection) [4]. In AI studies, a model is typically trained using a series of samples called a *train set*, which allows the model to understand the pattern that the information follows. The model’s performance is then tested using a *test set* that contains similar data to the train set but that the model has never seen before. In this way, the model is evaluated on previously unseen data that are different from the train set. It is common to use a portion of the train set as a *validation set* to study the effect of the model’s parameters (parameter tuning) and ensure that it generalizes well and does not simply memorize the train set (overfitting) [4,5,6].

In the context of user biometric authentication, both ML and DL have been shown to be useful tools. Both techniques have produced good results when using different biometric features, including: (1) behavioral characteristics with gyroscopes, accelerometers, and magnetometers [7,8], (2) physical attributes such as facial [9], ocular [10], or fingerprint [11] recognition, and (3) physiological signals such as electroencephalograms (EEG) [12] or electrocardiograms [13]. It is important to note the difference between the user identification problem and the user authentication problem. In the former, the model learns how to differentiate between multiple users, making it an MC problem with as many labels/classes as there are users. On the other hand, user authentication involves deciding whether a user is the legitimate one or not, which is a binary problem (positive data with label 1 for the legitimate user and negative data with label 0 for impostors). As a result, most AI-based user authentication systems employ MC (binary) classifiers, using positive training data from the user of interest and negative training data from other subjects. While this is a valid approach, it is not always reasonable to assume that information about impostors is available, and a more realistic scenario would involve using OC classifiers. In this case, the model would work as an anomaly detector, learning only from the information of the user of interest. However, this option typically lowers the efficiency of the authentication system [7].

EEG signals are one of the most unique physiological characteristics of an individual [14]. EEG is a test that records the bioelectrical brain activity of a subject using multiple sensors attached to the scalp. These signals are particularly useful for user authentication applications, as this biometric information provides enough intrapersonal consistency over time to identify a specific user, while still maintaining interpersonal differences that allow other individuals to be rejected [15]. By analyzing the frequency content of EEG signals, different brain waves can be extracted.

In this work, we introduce the use of Isolation Forest (IF) and Local Outlier Factor (LOF) models for user authentication based on EEG data, which, to our knowledge, has not been explored before. Additionally, we aim to construct a hybrid system that combines OC and MC classifiers to improve the performance of OC models using MC algorithms while only requiring information from the legitimate user. We also study different approaches to reduce the dimensionality of the problem. Since AI authentication systems are often time-consuming and computationally costly [7], it is useful to reduce the dimensions of the data used by the model. It is important to note that, since we are using EEG data, we are addressing the problem of user authentication, which is different from and more appropriate than the problem of device/smartphone authentication.

The rest of this article is organized as follows: in Section 2, we detail the contribution of this work, while in Section 3 the related work is described. In Section 4, the data acquisition and preprocessing are explained, while in Section 5 the methodology followed is detailed. Then, in Section 6, the produced results are presented, and in Section 7, we finish with some conclusions.

## 2. Article Contribution

In this paper, we investigate the use of different OC ML classifiers with EEG signals to develop secure and usable user authentication models and compare their performance with known successful MC ML classifiers. Our principal contribution is the introduction of the IF and LOF models for user authentication. Although they are well-known ML models, as far as we know, they have never been explored in this application. In this work, we show their suitability for OC EEG user authentication and improve the performance of similar works of the literature by studying the impact of their parameters in the security as well as in the usability aspects of the models. We consider that a model is *secure* if the probability of an authenticated user being the legitimate one is high (i.e., a precision above 90%), while a *usable* model would be the one in which the legitimate user requires a low number of attempts to be authenticated (i.e., a recall above 75%). Moreover, we aim to reduce the dimensionality of the problem and, hence, the computational costs of the system, without affecting the system performance. To do that, we will analyze the contribution of the information of each EEG channel and brainwave in the authentication process. Finally, we pretend to develop a hybrid protocol that combines an OC and an MC model that represents a realistic scenario and improves the results of the OC algorithm. Therefore, in this work, we address the following novelties:

- Solve the user authentication problem with IF and LOF, OC classifiers that have not been explored before for this application, increasing the literature authentication performance. We compared their results with OC–Support Vector Machine (OC–SVM) and, regarding MC classifiers, SVM, Random Forest (RF), and K-Nearest Neighbors (KNN). We have selected these models because they are well-known, classical ML models that have been shown to be useful in the user authentication application [7]. Moreover, as it will be exposed later, for each one of the MC models there is an equivalent OC model, which means that they have similar ways of working;
- Identify how the security and usability of the systems can be improved by modifying the parameters of the classifiers. As far as we know, this is the first work that presents this analysis regarding OC classifiers for EEG signals;
- Analyze the contribution to the authentication process of each channel and brain wave. We will reduce the dimensionality of the problem by selecting the most important channels and brain waves, and compare this dimensionality reduction methodology with Principal Component Analysis (PCA) and the statistical test $\chi^{2}$ [16];
- Construct a hybrid system that combines a OC and a MC model. In this sense, by using first an OC model, we will still be in a realistic scenario in which only the data of the legitimate user are needed, and then train a MC model by using the outputs of the OC model to improve the original results;
- The publication of the used scripts so that the experiments can be replicated with different databases (Script available at: https://github.com/luishalvarez/EEG-Authentication).

Our final goal is to implement the proposed mechanism in daily life situations by employing, for example, portable EEGs that can measure real-world signals in common and usual places [17]. Following this idea, our proposal could be applied for Continuous Authentication (CA), performing the authentication automatically and in a transparent manner. In order to do this, the ML model could be deployed in a smart device of the user, so that the EEG signals are directly sent to it. Another option could be to outsource the authentication to an external server in charge of managing, protecting, and processing the data. More evident applications are Brain–Computer Interfaces (BCI) and user authentication in hospitals. BCIs refer to systems that use the brain’s electrical activity to create a communications between a user and a computer to, for example, assist subjects with paralysis. Since these systems already measure the EEG, they could directly employ the proposed scheme to make sure that the communication includes the legitimate user, performing CA. In the case of hospitals, patients whose EEG must be acquired could be authenticated, ensuring that the correct medication/treatment is applied.

## 3. Related Work

We have divided the related works into two classes: user authentication with MC (Section 3.1) and OC (Section 3.2) classifiers. Additionally, in Section 3.3, we also include a brief discussion on works that have used IF and LOF with EEG data, but not for the application of user authentication.

### 3.1. Multi-Class EEG User Authentication

Several MC AI techniques have been studied and demonstrated to be useful regarding EEG-based user authentication. For instance, in [12], the authors employ an Artificial Neural Network (ANN) to authenticate users based on energy features extracted from the wavelet decomposition of their EEG signals. The reported results achieve the greatest values of 95% and 3.92% for True Positive Rate (TPR) and False Positive Rate (FPR), respectively, although their database only included five subjects. A comparable approach is followed in [18], where a Multilayer Perceptron (MLP) and a correlation model are utilized to identify the users, obtaining a final accuracy of 75.8%. Similar results were obtained with different configurations of ANNs in [19,20]. Convolutional Neural Networks (CNN) have also been studied in this field, as in [21,22,23], with results around 95% and 5% in accuracy and TPR.

The article included in [24] focused on the ML algorithms SVM, RF, KNN, and Naive Bayes. Positive outcomes were produced with all of them obtaining a greatest accuracy of 98.28% with an RF. These models have been analyzed with features extracted from the time domain [25] and the frequency domain [26]. Comparable results have been recently reported in [27,28]. Other suitable models include Hidden Markov Model (HMM) and Gaussian Mixture Models (GMM) [15].

### 3.2. One-Class EEG User Authentication

The research of OC classifiers for user authentication is based on the OC–SVM model. For example, a Support Vector Data Description (SVDD) is used in [29] to authenticate users based on their visual evoked potentials. The strategy consisted of combining the users in groups of three, and the result obtained was 98% of correctness. The accuracy (best value of 80%) and FPR (best value of 2.2%) of a OC–SVM were analyzed in [30] by increasing the number of blinks while measuring the EEG signals. In [31], the authors used an OC–SVM as a first security layer of an intruder detection/user identification system. In [32], OC–SVM and CNN were combined to construct a biometric authentication system. The study presented in [33] uses an OC–SVM to extract unsupervised features of EEG signals and explore their robustness against intra-subject variability.

### 3.3. IF and LOF with EEG Data

Recently, the combination of EEG signals with IF and LOF have been proposed in other works, but, to our knowledge, not for user authentication protocols. As an example, the study presented in [34] evaluates the use of IF to detect epileptic seizures in EEG signals. A similar objective is achieved in [35] utilizing fuzzy classifiers.

Similarly, in [36], outliers of eye state EEG signals were detected and eliminated by using IF. Alternatively, in [37], a pipeline for artifact removal in newborn EEG signals using LOF is introduced, employing it as an outlier detection algorithm. Approximated results are achieved in [38]. Another example of EEG outlier detection with LOF is [39], in which the EEG signals were used to classify breast tumors.

Based on the described literature, we can say that the IF and LOF models have not been explored for EEG-based user authentication. Even in other applications, the importance of IF and LOF was minimal compared to MC algorithms. However, in this work, we aim to study the suitability of these models to perform user authentication independently based on features already known to be successful. In the latest step, we pretend to build a hybrid system that improves their performance and only needs the information of the legitimate user.

## 4. Data Preparation

In this section, we describe how the EEG signals were acquired and the process followed to extract the features from which the AI models are going to learn.

### 4.1. Data Acquisition

**Figure 1:**
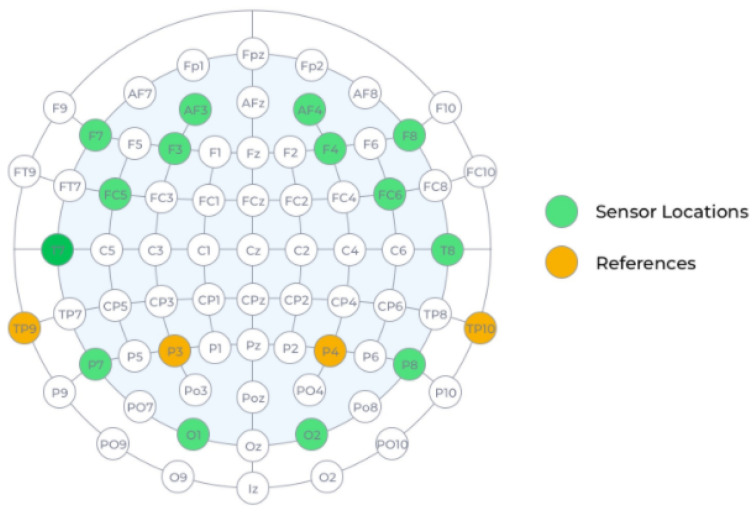
Measure map of Emotiv Epoc+ Headset [41].

To develop the measurement campaign, we took as reference the one described in [40]. The bioelectrical brain activity of 39 volunteers (23 women and 18 men between 18 and 60 years old) was measured. All volunteers were students or professors of the University of Florence and agreed to participate in the dataset collection only for research purposes. The signals were acquired with the *Emotiv Epoc+ V1.1* wireless headset [41] at a sampling rate of 256 Hz. This device is commercially available and consists of 18 contact sensors on flexible and fixed plastic arms that are placed on the scalp of the user. The device contains 14 EEG channels and four references that correspond to the following locations in the International 10–20 system: AF3, F7, F3, FC5, T7, P7, O1, O2, P8, T8, FC6, F4, F8, and AF4 for channels, and P3, P4, TP9, and TP10 for references [41]. In Figure 1, the location of the channels in a scalp is included. Additionally, the headset applies digital fifth-order sinc and 50 Hz notch filters that improve the quality of the signal acquired.

The measurements were conducted in an environment with controlled illumination and sonority, and without protection against electrical and electromagnetic interference. Before starting the experiment, the individuals were asked to sign an agreement, declaring that they did not suffer any form of neurological disorder, agreed to participate in the campaign, and allowed the use of their information for research purposes only by researchers of the University of Florence. Hence, due to ethical and privacy issues, the database used in this work is not publicly available. Once the headset was placed on the subjects, and it was verified that the signal was being measured successfully, the experiment started. Each session started with a black screen (5 s), followed by an image (20 s) and another black screen (5 s). As a focused state in the participant was desired, the image selected consisted of three bottles of wine with different and complex labels, so that the user had enough stimuli. This process was repeated in four different sessions with four different images during the same day for each user. The four EEG signals were then joined, forming a single EEG signal for each user with an approximated duration of 2 min.

### 4.2. Data Preprocessing

For each subject *i*, 14 continuous EEG signals (one signal per each channel *c*) over time *t*, $S_{i,c}\left(t\right)$ were obtained. It is important to notice that signals of different users were not recorded for the same exact time interval and, therefore, the length of the signals varies from user to user. We defined the following data preprocessing procedure:

1. We divided the EEG signal of each channel and user in smaller signals of 240 ms. In other words, each $S_{i,c}\left(t\right)$ is separated in $j_{i}$ smaller signals, $s_{i,c,j_{i}}\left(t\right)$ of 240 ms each. These smaller signals $s_{i,c,j_{i}}\left(t\right)$ represent different samples of the same subject *i*. We chose 240 ms as time interval for two reasons: (1) it was viable to extract the frequency content (i.e., the brainwaves) we are interested in, and (2) a sufficient number of samples for each user were obtained. Since signals of different users had a different duration, the number of smaller signals *j* is different for each user (from here the notation $j_{i}$), and it goes from 1 to $m_{i}$, where $m_{i}$ is the total number of smaller signals for the user *i*.
2. Then, we computed the wavelet decomposition of each $s_{i,c,j_{i}}\left(t\right)$ for i∈{1,…,39}, c∈{1,…,14}, and $j_{i}\in \left\{1,\ldots ,m_{i}\right\}$. Specifically, we employed a five-level wavelet decomposition using the order 2 Daubechies wavelet with Matlab, version R2021b. From this decomposition, we acquired the wavelet coefficients D1, D2, D3, D4, D5, and A5 as real-valued vectors. In Table 1, the frequency content and the corresponding brain wave of each coefficient are reported.
  We decided to use the wavelet decomposition for several reasons: (i) the implementation of the Fast Wavelet Transform is computationally fast, (ii) it offers a simultaneous signal feature localization in time and frequency domain, (iii) it is able to identify details of small parts of the signal, better than its general characteristics, and (iv) it has been shown to be suitable to an AI model with EEG data [25].
3. Finally, for each wavelet coefficient vector D1, D2, D3, D4, D5, and A5 in each $s_{i,j_{i}}\left(t\right)$, we calculated the following eight metrics [25]: maximum, minimum, mean, standard deviation, variance, skewness, Shannon entropy, and average power.

Taking into account that the *Emotiv Epoc+* headset acquires a signal from each of its 14 channels that six coefficients are calculated for each signal, eight metrics are computed for each wavelet coefficient, and the final number of features is 14×6×8=672.

## 5. Methodology

In this section, we explain the authentication strategy, which includes how the AI models are going to be trained and tested, and provide a brief explanation of the models that we will use, as well as of their more important parameters. Additionally, we include a threat model of our proposal.

### 5.1. Authentication Strategy

ML algorithms learn from data whose features have to be defined manually. In the previous section, we described how we conducted this procedure. Additionally, as already explained, we are going to divide the data into a train set, a validation set, and a test set to improve the model as much as possible and avoid overfitting.

A user authentication protocol is composed by two phases: the first one is the *Enrollment Phase*, in which data from the user of interest are collected and used to train a model. Once the model is constructed, the user can be authenticated by introducing a new measurement; this corresponds to the *Authentication Phase*. We associate the Enrollment Phase with the model training, whereas the model testing represents the Authentication Phase. To execute each phase, we differentiate between OC and MC classifiers:

- Multi-Class classifiers: they need both positive (from the legitimate user) and negative (from an impostor) samples to be trained. In our case, the positive samples of the train set are the 80% of the total samples of the legitimate user, and the remaining 20% is used for testing. In addition, 15% of the train set represents the validation set. The negatives samples are randomly selected from the other 38 subjects for all sets, and the proportion of positive and negative samples in each set is defined as 50%.
- One-Class classifiers: in this case, the train set consists of 80% of the total samples of the legitimate user, as these models only need positive samples. The test set is composed of the remaining 20% (positive samples), and the same number of negative samples, randomly selected from the other 38 users.

It should be mentioned that, in both cases, the data are normalized attending to the standard normalization.

### 5.2. Classifiers and Dimensionality Reduction

As described, both MC and OC ML classifiers have been used to develop user authentication mechanisms based on EEG data. On the one hand, MC models usually achieve better performances, as they learn the information structure of both the legitimate user and a potential impostor. Nevertheless, the information of the impostor is commonly represented by randomly selected data from the remaining subjects in the database. On the other hand, despite OC classifiers not being as efficient, they only need the information of the legitimate user to be trained.

Below, we describe the MC models proposed.

SVM: A model that constructs a hyperplane in a high-dimensional space to separate two set of points (classes). To find the optimal hyperplane, SVMs maximize its distance to the nearest element of each class, called the functional margin. We employ a Radial Basis Function (RBF) kernel function to simplify the calculations of the SVM, defining a 10-fold cross–validation process to optimize the value of the parameters *C* and *gamma*. The parameter *C* indicates how small the margin hyperplane is, while *gamma* defines the relevance of each training sample [1,4,5]. The specific values studied in the cross–validation process were C={0.1,1,10,100,1000} and *gamma* = {*auto*, *scale*, 0.001,0.01,0.1,1,10,100} [5].

RF: Bagging method that combines several individual decision tree classifiers [5], and whose result is the most voted class. Decision tree classifiers are models in which the leaves represent features, while branches represent labels. In this sense, the model learns how to differentiate the labels depending on the value of the features, hence creating new branches from each leave. In this work, we construct ensembles of 500 decision tree classifiers with default maximum depth (number of leaves and branches) [4,5].

KNN: It predicts the label of a new sample by looking at the most voted label of the *k* nearest known samples. In this sense, several metrics can be used to define distance, as, for example, the Euclidean distance of the features. In our case, we will set the number of neighbors to 25 (k=25) and use the default values for the rest of parameters [4,5].

The OC classifiers evaluated are the following:

OC–SVM: Schölkopf et al. [42] proposed a similar technique to SVM to construct a hyperplane that separate regions with and without data, called OC–SVM. Thus, OC–SVM has the same parameters of SVMs but also include the ν parameter. This new parameter is both a lower bound for the number of samples that are support vectors and an upper bound for the number of samples that are on the wrong side of the hyperplane. This is a difficult parameter to cross–validate and, certainly, we are going to explore its effect in the security and usability of the model [43].

IF: This algorithm detects anomalies by individually isolating points with binary tree classifiers, instead of modeling the normal points. This fact implies that IFs are a fast anomaly detector, with linear time complexity. Its parameters are the ones of RF and, consequently, we will use 500 estimators. Additionally, its *contamination* parameter is the analogous to the ν parameter of OC–SVM and, hence, we will analyze it also in this case [44].

LOF: Proposed by Breunig et al. [45], LOFs detect anomalous samples by measuring the local deviation or local density of each point with respect to its neighbors. Hence, this model is similar to KNN, but including the “contamination” parameter, which has the same meaning as for IF and OC–SVM [44].

From the included classifiers’ descriptions, it can be inferred that an OC model corresponds to each MC model (and vice versa). That is, the pairs SVM and OC–SVM, RF and IF, and KNN and LOF have approximated methodologies and, despite the fact that there are some important differences, their basic functioning is similar. As a result, we expect the best individual models (and, thus, the models of the hybrid system) to be one of these pairs.

As stated in Section 4.2, the initial number of features of our data are 672. It would be interesting to reduce this number, so that the models could be more computationally efficient (i.e., consume less time), while avoiding the reduction in their performances. We will explore the following dimensionality reduction techniques:

PCA: New features are going to be defined as linear combinations of the original ones. In this study, 32 and 64 new features were obtained with PCA, as we found that their representation percentage was sufficient (see Section 6.2).

Statistical Test $\chi^{2}$: This test enables us to identify the most significant original features. We will evaluate the models considering only the 32 and 64 most significant features (see Section 6.2).

Channel/brainwave: Alternatively, to reduce the problem dimensionality, we propose the study of each EEG channel and brainwave individually to test their contribution to the authentication model. Each channel contains 6×8=48 features, and each brainwave 14×8=112 features. Thus, detecting the most representative channels and brainwaves would allow us not only to reduce the dimensions of the problem, but also to extract the biological meaning.

### 5.3. Threat Model

Several security requirements should be pinpointed in the suggested authentication methodology. Considering that, in general, the user’s information is outsourced to an external server, the first security concern is the preservation of the user’s privacy. This means that protocols that guarantee data confidentiality, integrity, availability, and non–repudiation should be implemented. In addition, all parties that participate in the protocol should be authenticated before it is started. These needs can be established by defining a secure channel for data transmission and employing symmetric key cryptosystems. In fact, other works have studied the user authentication problem by using encrypted data as input to AI models [7], so that the clear information does not need to leave the sensor. Alternatively, although the general authentication protocol (data acquisition and preprocessing, and the ML models used) should be public, the specific parameters of the preprocessing step and models should be secret. By doing this, if an attacker has access to the user’s data, he will not be able to construct a new model that can imitate the original one.

The fulfillment of these requirements is crucial to avoid dataset poisoning attacks, model substitution attacks [46], stealth attacks [47], and membership inference attacks [48], among others [49]. Apart from this, an adversary may attack the protocol itself, trying to fool the model with false data. In this case, we distinguish two scenarios [50]:

- Random or substitution attack: an adversary uses his own EEG signal to be (incorrectly) authenticated. As described, our models are tested by using positive and negative samples. Therefore, we are already considering and overcoming this attack.
- Skilled forgery attack: an adversary tries to reproduce the user’s EEG signal as closely as possible to be (incorrectly) authenticated. The execution of this attack is more evident with authentication systems based on gestures; the attacker tries to reproduce the legit user’s movement. However, EEG signals cannot be imitated so easily. Consequently, we think that the best approximation for this attack is to train a Generative Adversarial Network (GAN) that generates similar EEG signals to the real user. Despite this attack not being addressed in the presented work, it is our objective to work on it shortly.

Lastly, we should also consider that there might be different factors affecting the acquisition of the EEG signals, thus decreasing the performance of the protocol. Some examples of them might be a malfunctioning sensor, software failures, or undesired user conditions (e.g., stress, intoxication, etc.).

### 5.4. Results Acquisition

As explained before, we aim to compare the performance of OC and MC ML classifiers (while evaluating the effect of dimensionality reduction techniques), the influence of each channel and brainwave, and the impact of the ν/*contamination* parameter related to the security and usability of the authentication system. To do that meaningfully, the following steps are followed:

1. Firstly, we carry out some baseline results with the MC and OC algorithms and compare their performance using the whole set of features (Section 6.1);
2. Then, we compare the baseline results with the outcomes after applying the dimensionality reduction procedures PCA and $\chi^{2}$, decreasing the dimensions to 32 and 64 in both cases (Section 6.2);
3. Next, the contribution of each channel and brainwave is evaluated by solving the authentication problem with just the data of each channel/brainwave (Section 6.3);
4. After that, we evaluate the effect of the ν and “contamination” parameter in the OC classifiers in order to obtain more secure/usable authentication systems (Section 6.4);
5. Finally, taking into account the obtained results, we explore the most promising combinations of OC and MC classifiers using the data with greater contribution in the authentication problem (Section 6.5).

These comparisons are made attending to different metrics: precision, recall, F_1_-score, accuracy, and FPR. All of them are based on the number of True Positives (TP), False Negatives (FN), False Positives (FP), and True Negatives (TN) of each case. The definition of the metrics are included in the following formulas:

$$\mathrm{Precision}=\frac{\mathrm{TP}}{\mathrm{TP}+\mathrm{FP}},\;\mathrm{Recall}=\frac{\mathrm{TP}}{\mathrm{TP}+\mathrm{FN}},$$

$$F_{1}--\mathrm{Score}=2\frac{\mathrm{Precision}\cdot \mathrm{Recall}}{\mathrm{Precision}+\mathrm{Recall}},$$

$$\mathrm{Accuracy}=\frac{\mathrm{TP}+\mathrm{TN}}{\mathrm{TP}+\mathrm{FP}+\mathrm{TN}+\mathrm{FN}},\;\mathrm{FPR}=\frac{\mathrm{FP}}{\mathrm{FP}+\mathrm{TN}}$$

According to these definitions, we could associate the precision (percentage of times a predicted positive was a true positive) and FPR (percentage of times an impostor was identified as the owner from his total number of attempts) with the security of the model. In other words, we can say that our model is secure if it shows a high precision (above 90%) and a low FPR (less than 10%). On the contrary, the usability of the system is directly proportional to the recall (percentage of times the owner is correctly authenticated). The F_1_-score is the harmonic average of precision and recall, which means that it can be analyzed to identify balanced systems. Finally, the accuracy can give us an insight into how well the model performs, and if there is something undesired happening (i.e., a constant accuracy of 0.5 would mean that the model is either rejecting or accepting all samples and, therefore, is not generalizing).

Apart from the above measures, we are also going to use the Area Under the Receiver Operating Characteristic (ROC) Curve (AUC) and the Equal Error Rate (EER) to evaluate the MC classifiers. The axes of the ROC curve are typically false positive rate (x-axis) and true positive rate (y-axis) and the greater its area, the better the performance. On the contrary, a smaller EER is associated with higher accuracies, and it is defined as the point of the ROC curve in which the false acceptance rate and false rejection rate are equal. However, these two metrics are obtained from probabilities and, since the OC classifiers directly accept or reject a sample, their use does not make sense with OC models.

## 6. Results

In this section, we include the outcomes obtained following the procedure described previously.

### 6.1. Baseline OC and MC—All Features

The first step is to obtain the baseline results, shown in Table 2. These results represent, for each metric, the average of the 39 users, and were obtained with the total number of features (672).

An important consideration is that, since the negative samples were randomly selected from the data of the remaining users, we repeated each experiment three times. In any case, the standard deviation of the outcomes obtained was higher than 0.02. We do not include this information in the tables to facilitate the results’ visualization to the reader, but the data justify that the randomization does not affect the performance of the models.

If we compare the MC models, the SVM and RF offer better security (greater precision and FPR values) and accuracy than KNN, although their usability (recall) are approximated.

With respect to the OC classifiers, although they maintain comparable results with MC in terms of security (specially in the case of IF), they experience a dramatic reduction in usability and accuracy. This means that it is more difficult to balance the security–usability relation for OC models and, thus, they are either severe or usable. Interestingly, IF and LOF present similar accuracy values, but LOF shows a more equilibrated security–usability balance.

**Figure 2:**
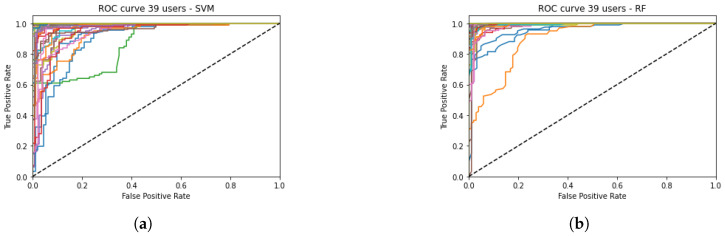
ROC Curves of the 39 users (colored lines) with 672 features for SVM (**a**) and RF (**b**).

In Figure 2a,b, the ROC curves of the 39 users for the SVM and RF are respectively represented. It can be appreciated that the curves behave similarly for every subject, with the exception of two users in the case of SVM (green and orange curves) and one user in the case of RF (orange curve). This leads us to think that the configuration of the models is appropriated.

### 6.2. Dimensionality Reduction

We applied the traditional dimensionality reduction techniques PCA and statistical test $\chi^{2}$ and performed the same tests as in the previous section. We analyzed the results produced by reducing the dimensions to 8, 16, 32, 64, and 128. However, we observed that, for PCA, the results improved as the number of features increased until there were 32, the point at which the performance started to deteriorate. In the case of $\chi^{2}$, the results are better with a higher number of features, but, for consistency, we decided to maintain the same number of features in both methods. As a result, Table 3 includes the outcomes obtained with 32 (best case for PCA) and 64 features. In both cases, the representation of the new features was greater than 99.999%.

Comparing the results with the ones presented in Section 6.1, we can see that the performances of the models have become worse, more abruptly for the OC classifiers. RF and SVM are still working remarkably better than KNN and, in light of these results, we decided to focus on SVM and RF for the remaining experiments, discarding KNN.

Looking at each procedure individually, it can be inferred that $\chi^{2}$ has more troubles when working with MC models than PCA, but they perform similarly with OC classifiers. RF remains the best MC model in both cases and, for the OC ones, LOF is the best for PCA and IF for $\chi^{2}$.

### 6.3. Channel and Wave Comparison

Now, we analyze the user authentication problem taking into account each channel and brainwave individually. In Table 4 and Table 5, the results obtained for the channels and brainwaves in the case of MC classifiers are respectively presented. The same information regarding the OC models is included in Table 6 and Table 7.

For SVM and RF, the results suggest that the most important channels are P8, T8, FC6, and F4. Additionally, channel AF3 also contributes to the security for the RF. For the case of brainwaves, both models coincide, with gamma (D1 and D2) and beta (D3) waves as the most influential ones. It should be noticed that, despite the classifiers coinciding in the importance of channels and waves, the results are always better with RF. In other words, for the same channel or the same brainwave, the metrics present better values for RF than for SVM. This leads us to think that RF should be a preferred option.

With respect to the OC models, the channels that contribute the most are P8, T8, FC6, and F4, coinciding P8, FC6, and F4 as the ones with better security and usability for OC–SVM and IF. Moreover, the best result of each individual channel is always obtained with IF. Nevertheless, they present more distributed results regarding the brainwaves and, hence, it is not clear which is the best option. For high frequency components (the best choices for MC models), it looks like IF works better, whereas LOF performs the best for low frequency components.

### 6.4. Effect of Contamination

We want to identify the effect of the ν and *contamination* parameters in the performance of the models. To do that, we explored a set of different values for both parameters, {0.01,0.03,0.05,0.1,0.3,0.5} for each one of the OC classifiers using the 112 features of D1 (all channels). In Table 8, these results are included.

For all classifiers, the security is proportional to the parameters studied, while the usability is inversely proportional. This means that, for the value 0.5, the highest security level is obtained, and for 0.01 the greatest usability level is reached. For intermediate values, the relation between security and usability is more balanced, and Table 8 suggests that the value 0.3 achieves the best equilibrium.

### 6.5. OC and MC Combination

Finally, we propose a hybrid system that combines an OC with an MC classifier. To maintain a realistic scenario, the first layer of the system is the OC model, trained with only positive data, as data from other users, including malicious ones, are not typically available. The test data of this model will then be introduced as train data to a MC classifier, labeled by the OC model. In this way, we manage to improve the results of OC models by the action of the MC classifier.

Consequently with the results presented in the previous sections, we decided to select IF and RF as the OC and MC classifiers of the described system, respectively. They were the ones that presented the best outcomes and match in their most significant channels. To test the system, we have defined several configurations depending on the data used to train the first model. All the configurations and the results are showed in Table 9. In order to understand the importance of these results, we have included the outcomes obtained using all data (Complete), and the data reduced with PCA and $\chi^{2}$. It should be clarified that we settled the value of the parameter *contamination* to 0.3 in all cases.

We can appreciate that the best results are obtained with Configuration III, in which the features are formed by D1 coefficient (gamma wave) of all channels. Hence, it seems that this option is preferable over using a combination of brainwaves and less channels. Actually, the results obtained using all the features are worse, which leads us to think that we are using useful features and avoiding non-representative information. We can consider that Configuration III has an appropriated level of security (91.2% of precision and 8.4% of FPR) and a good level of usability (76.0% of recall). In addition, we added a column to include the mean time (in seconds) that it takes to train a model. It should be clarified that all experiments have been carried out on Intel Core i7 at 2.00 GHz and 16 GB of RAM, using Python 3.9.

It can be seen that the greater the number of features, the larger time the model takes. Certainly, each model takes 2 s to be trained when all features are used and, in the configuration proposed, we maintain this time to less than 1 second (49.23% of reduction). This time is less than 0.1 s larger than the one obtained with Configuration I (the lowest obtained). As a result, we consider Configuration III the best option among the studied ones.

To guarantee that the results presented are coherent independently of the initial data, and as a double-check for our proposal, we repeated the experiments with Configuration III modifying the train and test sets. In other words, we randomly permuted the samples of all users five times and solved the authentication problem for each case. The results are presented in Table 10, and the stability of the results can be easily appreciated.

**Figure 3:**
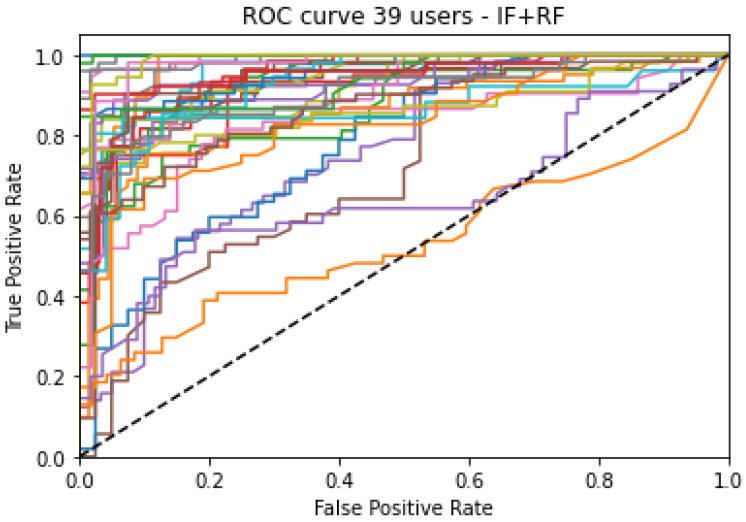
ROC Curves of the 39 users (colored lines) for configuration III.

From the biological point of view, D1 represents high gamma waves, which are related to intense brain activities and cognitive phenomena such as attention. Taking into account the nature of the experiments performed to obtained the EEG signals, the preferable use of D1 over the rest of brainwaves makes sense. In Figure 3, the ROC curve of Configuration III can be found. It is easy to see that all users behave similarly, with the exception of two of them.

## 7. Conclusions

The development of secure and user-friendly authentication systems is essential in today’s world, where many routines and activities are based on online services. The security of these systems protects user information, while their usability enhances the user experience.

In this work, we have investigated the use of OC ML classifiers to solve the problem of user authentication individually and in combination with MC models. The authentication system was based on EEG data that we collected in an experimental campaign, as it is considered one of the most unique and characteristic features of an individual’s body. Therefore, the proposed system does not recognize the user’s device, but the specific user. We also identified the channels and brainwaves that contribute most to distinguishing a user, finding that high-frequency content (i.e., high brain activity information) provides the most significant information. Interestingly, the results suggest that Isolation Forest (IF), an unexplored OC model in this application, is the best option. Furthermore, we propose a hybrid system using IF and RF classifiers, which achieved an accuracy of 82.3%, a precision of 91.1%, a false positive rate (FPR) of 8.3%, and a recall of 75.3%.

In future research, we aim to improve these results by combining EEG signals with other biometric features such as galvanic skin response (GSR), eye movement, or information from inertial sensors (e.g., accelerometers and gyroscopes). We also plan to explore the use of DL techniques (one-class ANN, CNN) to solve the user authentication problem and compare the results of ML and DL. Finally, we intend to train a GAN and simulate skilled forgery attacks for EEG user authentication.

## Figures and Tables

**Table 1:** Correspondence between Wavelet Coefficients, Brain Waves, and Frequency Content.

**Wavelet Coeff.**	D1	D2	D3	D4	D5	A5
**Brain wave**	γ	γ	β	α	θ	δ
**Freq. (Hz)**	64–128	32–64	16–32	8–16	4–8	0–4

**Table 2:** Results with 672 features.

No Dim. Red.	AUC	EER	Prec.	Recall	$F_{1}$	Acc	FPR
SVM	0.980	0.056	0.943	0.955	0.948	0.948	0.059
RF	0.991	0.033	0.970	0.957	0.962	0.964	0.029
KNN	0.899	0.157	0.775	0.905	0.823	0.802	0.301
OC–SVM	-	-	0.871	0.547	0.652	0.730	0.087
IF	-	-	0.910	0.537	0.663	0.741	0.054
LOF	-	-	0.821	0.621	0.691	0.730	0.160

**Table 3:** Results with PCA and $\chi^{2}$—32 and 64 features.

	PCA								$\chi^{2}$						
	AUC	EER	Prec.	Recall	$F_{1}$	Acc	FPR		AUC	EER	Prec.	Recall	$F_{1}$	Acc	FPR
	32 Features														
**SVM**	0.887	0.176	0.834	0.780	0.797	0.814	0.152		0.752	0.300	0.682	0.725	0.690	0.690	0.344
**RF**	0.967	0.087	0.911	0.898	0.900	0.906	0.087		0.781	0.285	0.707	0.748	0.723	0.718	0.312
**KNN**	0.794	0.269	0.722	0.635	0.648	0.681	0.273		0.683	0.363	0.636	0.616	0.610	0.632	0.352
**OC–SVM**	-	-	0.624	0.525	0.547	0.589	0.346		-	-	0.633	0.568	0.587	0.606	0.355
**IF**	-	-	0.624	0.527	0.547	0.588	0.351		-	-	0.649	0.551	0.581	0.613	0.326
**LOF**	-	-	0.705	0.627	0.651	0.669	0.290		-	-	0.585	0.579	0.575	0.570	0.439
	**64 Features**														
**SVM**	0.909	0.161	0.818	0.853	0.823	0.828	0.198		0.811	0.251	0.733	0.793	0.756	0.747	0.299
**RF**	0.962	0.090	0.901	0.898	0.893	0.900	0.098		0.845	0.223	0.768	0.807	0.784	0.779	0.248
**KNN**	0.777	0.262	0.727	0.559	0.587	0.652	0.255		0.736	0.302	0.687	0.689	0.673	0.680	0.329
**OC–SVM**	-	-	0.592	0.511	0.527	0.565	0.381		-	-	0.662	0.571	0.599	0.623	0.324
**IF**	-	-	0.571	0.510	0.520	0.552	0.407		-	-	0.713	0.545	0.604	0.651	0.242
**LOF**	-	-	0.658	0.609	0.620	0.632	0.345		-	-	0.616	0.594	0.600	0.603	0.388

**Table 4:** Results per Channel with MC—48 features.

	SVM								RF						
	AUC	EER	Prec.	Recall	$F_{1}$	Acc	FPR		AUC	EER	Prec.	Recall	$F_{1}$	Acc	FPR
**AF3**	0.789	0.277	0.712	0.781	0.743	0.732	0.316		0.833	0.233	0.756	0.801	0.777	0.771	0.258
**F7**	0.756	0.296	0.688	0.766	0.720	0.705	0.356		0.794	0.266	0.720	0.776	0.745	0.736	0.300
**F3**	0.795	0.268	0.713	0.790	0.745	0.734	0.323		0.828	0.238	0.751	0.798	0.771	0.764	0.270
**FC5**	0.801	0.260	0.718	0.808	0.756	0.743	0.321		0.833	0.237	0.747	0.806	0.773	0.766	0.274
**T7**	0.757	0.301	0.688	0.765	0.718	0.707	0.351		0.799	0.275	0.717	0.753	0.732	0.732	0.290
**P7**	0.781	0.283	0.707	0.817	0.752	0.734	0.348		0.828	0.242	0.743	0.802	0.767	0.760	0.281
**01**	0.788	0.272	0.710	0.803	0.748	0.734	0.336		0.830	0.239	0.748	0.810	0.776	0.769	0.273
**02**	0.782	0.275	0.714	0.778	0.737	0.726	0.326		0.836	0.236	0.756	0.804	0.777	0.769	0.267
**P8**	0.821	0.240	0.742	0.819	0.774	0.766	0.287		0.863	0.208	0.780	0.840	0.807	0.800	0.240
**T8**	0.802	0.252	0.727	0.818	0.766	0.752	0.314		0.839	0.226	0.755	0.809	0.779	0.774	0.262
**FC6**	0.792	0.273	0.716	0.823	0.762	0.741	0.340		0.824	0.244	0.742	0.819	0.776	0.764	0.292
**F4**	0.804	0.248	0.740	0.785	0.754	0.755	0.275		0.868	0.194	0.801	0.826	0.809	0.809	0.208
**F8**	0.766	0.294	0.695	0.768	0.724	0.708	0.353		0.792	0.267	0.726	0.766	0.743	0.736	0.295
**AF4**	0.766	0.288	0.700	0.775	0.730	0.716	0.344		0.820	0.241	0.748	0.779	0.759	0.757	0.264

**Table 5:** Results per Wave with MC—112 features.

	SVM								RF						
	AUC	EER	Prec.	Recall	$F_{1}$	Acc	FPR		AUC	EER	Prec.	Recall	$F_{1}$	Acc	FPR
**D1**	0.969	0.065	0.927	0.956	0.940	0.939	0.079		0.985	0.044	0.955	0.947	0.950	0.951	0.044
**D2**	0.962	0.071	0.927	0.935	0.929	0.930	0.074		0.980	0.055	0.948	0.931	0.938	0.940	0.051
**D3**	0.969	0.066	0.927	0.943	0.935	0.934	0.075		0.985	0.048	0.956	0.935	0.945	0.946	0.042
**D4**	0.955	0.088	0.900	0.922	0.909	0.907	0.108		0.973	0.070	0.935	0.927	0.930	0.931	0.066
**D5**	0.943	0.114	0.870	0.910	0.888	0.885	0.141		0.958	0.095	0.914	0.893	0.902	0.903	0.087
**A5**	0.942	0.109	0.889	0.895	0.889	0.891	0.113		0.973	0.075	0.930	0.908	0.916	0.919	0.069

**Table 6:** Results per Channel with OC—48 features.

	OC–SVM (ν = 0.5)						IF (contamination = 0.5)						LOF (contamination = 0.5)				
	Prec.	Recall	$F_{1}$	Acc	FPR		Prec.	Recall	$F_{1}$	Acc	FPR		Prec.	Recall	$F_{1}$	Acc	FPR
**AF3**	0.627	0.512	0.556	0.594	0.325		0.684	0.498	0.570	0.630	0.238		0.583	0.542	0.557	0.564	0.413
**F7**	0.615	0.537	0.566	0.588	0.362		0.660	0.508	0.568	0.615	0.277		0.580	0.537	0.555	0.566	0.406
**F3**	0.623	0.542	0.571	0.596	0.350		0.665	0.519	0.575	0.620	0.279		0.578	0.555	0.560	0.560	0.435
**FC5**	0.612	0.523	0.557	0.585	0.352		0.662	0.518	0.572	0.618	0.283		0.576	0.543	0.554	0.554	0.435
**T7**	0.615	0.494	0.540	0.585	0.325		0.636	0.467	0.532	0.596	0.276		0.577	0.517	0.541	0.559	0.400
**P7**	0.619	0.530	0.563	0.595	0.339		0.659	0.494	0.557	0.615	0.263		0.559	0.546	0.549	0.547	0.453
**01**	0.628	0.526	0.561	0.593	0.340		0.678	0.518	0.579	0.630	0.257		0.568	0.539	0.548	0.548	0.443
**02**	0.659	0.558	0.589	0.617	0.323		0.719	0.532	0.599	0.654	0.225		0.578	0.571	0.566	0.560	0.450
**P8**	0.687	0.563	0.606	0.639	0.285		0.748	0.541	0.619	0.672	0.197		0.598	0.567	0.573	0.571	0.425
**T8**	0.652	0.547	0.584	0.623	0.301		0.711	0.537	0.603	0.660	0.218		0.587	0.572	0.575	0.577	0.418
**FC6**	0.661	0.570	0.605	0.632	0.306		0.716	0.550	0.616	0.663	0.224		0.576	0.576	0.571	0.564	0.448
**F4**	0.717	0.560	0.617	0.654	0.252		0.755	0.552	0.627	0.678	0.196		0.602	0.572	0.579	0.578	0.416
**F8**	0.612	0.560	0.576	0.588	0.385		0.644	0.538	0.577	0.609	0.319		0.566	0.556	0.558	0.559	0.439
**AF4**	0.631	0.558	0.578	0.596	0.366		0.687	0.540	0.592	0.635	0.270		0.584	0.581	0.578	0.574	0.433

**Table 7:** Results per Wave with OC—112 features.

	OC–SVM (ν = 0.5)						IF (Contamination = 0.5)						LOF (Contamination = 0.5)				
	Prec.	Recall	$F_{1}$	Acc	FPR		Prec.	Recall	$F_{1}$	Acc	FPR		Prec.	Recall	$F_{1}$	Acc	FPR
**D1**	0.774	0.524	0.615	0.674	0.176		0.947	0.510	0.655	0.740	0.030		0.705	0.570	0.624	0.657	0.255
**D2**	0.839	0.507	0.622	0.699	0.109		0.945	0.486	0.635	0.729	0.029		0.767	0.579	0.653	0.695	0.190
**D3**	0.843	0.502	0.622	0.700	0.102		0.925	0.488	0.633	0.724	0.041		0.783	0.572	0.655	0.701	0.171
**D4**	0.798	0.510	0.617	0.687	0.136		0.860	0.507	0.634	0.710	0.087		0.781	0.587	0.665	0.705	0.176
**D5**	0.744	0.541	0.621	0.673	0.194		0.778	0.537	0.627	0.688	0.161		0.743	0.604	0.662	0.693	0.219
**A5**	0.677	0.555	0.593	0.639	0.278		0.678	0.562	0.597	0.638	0.286		0.759	0.633	0.681	0.711	0.210

**Table 8:** Results for Different Values of ν and contamination with OC—112 features (D1).

	OC–SVM (D1)						IF (D1)						LOF (D1)				
	Prec.	Recall	$F_{1}$	Acc	FPR		Prec.	Recall	$F_{1}$	Acc	FPR		Prec.	Recall	$F_{1}$	Acc	FPR
**0.5**	0.772	0.524	0.612	0.671	0.182		0.940	0.513	0.658	0.741	0.030		0.701	0.569	0.622	0.656	0.257
**0.3**	0.702	0.723	0.701	0.688	0.347		0.875	0.717	0.781	0.804	0.110		0.638	0.772	0.696	0.663	0.446
**0.1**	0.626	0.900	0.729	0.657	0.585		0.712	0.911	0.791	0.754	0.404		0.562	0.945	0.705	0.604	0.738
**0.05**	0.604	0.938	0.726	0.638	0.663		0.633	0.955	0.756	0.686	0.583		0.535	0.980	0.692	0.564	0.853
**0.03**	0.593	0.945	0.721	0.626	0.694		0.580	0.975	0.725	0.624	0.726		0.523	0.991	0.685	0.544	0.903
**0.01**	0.602	0.947	0.728	0.636	0.675		0.509	0.995	0.673	0.516	0.962		0.510	0.999	0.675	0.520	0.959

**Table 9:** Best OC-MC configurations.

Configuration	#Features	Waves	Channels	AUC	EER	Prec.	Recall	$F_{1}$	Acc	FPR	t (s)
I	40	D1	AF3, P8, T8, FC6, F4	0.866	0.198	0.842	0.752	0.785	0.789	0.172	0.903
II	64	D1	AF3, 01, 02, P8, T8, FC6, F4	0.894	0.174	0.892	0.756	0.806	0.815	0.121	0.927
III	112	D1	all	0.907	0.147	0.912	0.760	0.829	0.834	0.084	0.996
IV	80	D1,D2	AF3, P8, T8, FC6, F4	0.893	0.168	0.880	0.751	0.798	0.810	0.126	0.960
V	120	D1,D2,D3	AF3, P8, T8, FC6, F4	0.888	0.172	0.870	0.745	0.782	0.798	0.141	1.005
Complete	672	all	all	0.899	0.153	0.875	0.766	0.786	0.804	0.151	2.023
Best PCA	32	-	-	0.627	0.387	0.628	0.757	0.661	0.615	0.541	0.906
Best $\chi^{2}$	32	-	-	0.673	0.375	0.636	0.794	0.694	0.635	0.539	0.909

**Table 10:** Five different permutations of Configuration III.

Permutation	Configuration	AUC	EER	Prec.	Recall	$F_{1}$	Acc	FPR	t (s)
1	III	0.907	0.147	0.912	0.760	0.829	0.834	0.084	0.996
2	III	0.904	0.163	0.924	0.727	0.814	0.815	0.076	0.998
3	III	0.900	0.163	0.900	0.762	0.825	0.803	0.096	0.997
4	III	0.912	0.140	0.915	0.771	0.837	0.849	0.070	0.996
5	III	0.902	0.151	0.904	0.43	0.816	0.813	0.088	0.999
Average	III	0.905	0.153	0.911	0.753	0.824	0.823	0.083	0.997
Standard Deviation	III	0.005	0.010	0.009	0.018	0.010	0.018	0.010	0.001

## Data Availability

Not applicable.

## Abbreviations

The following abbreviations are used in this manuscript:

AI	Artificial Intelligence
ANN	Artificial Neural Network
AUC	Area Under the Curve
BCI	Brain Computer Interface
CA	Continuous Authentication
CNN	Convolutional Neural Network
DL	Deep Learning
EEE	Electroencephalogram
EER	Equal Error Rate
FPR	False Positive Rate
GMM	Gaussian Mixture Model
GSR	Galvanic Skin Response
HMM	Hidden Markov Model
IF	Isolation Forest
KNN	K-Nearest Neighbors
LOF	Local Outlier Factor
MC	Multi-Class
ML	Machine Learning
MLP	Multilayer Perceptron
OC	One-Class
PCA	Principal Component Analysis

RF	Random Forest
ROC	Receiver Operating Characteristic
SVDD	Support Vector Data Description
SVM	Support Vector Machine
TPR	True Positive Rate
